# Interfacial chemical bonding-mediated ionic resistive switching

**DOI:** 10.1038/s41598-017-01493-x

**Published:** 2017-04-28

**Authors:** Hyeongjoo Moon, Vishal Zade, Hung-Sen Kang, Jin-Woo Han, Eunseok Lee, Cheol Seong Hwang, Min Hwan Lee

**Affiliations:** 1 School of Engineering, University of California, Merced, CA 95343 USA; 2 Center for Nanotechnology, NASA Ames Research Center, Moffett Field, CA 94035 USA; 3 Department of Mechanical and Aerospace Engineering, University of Alabama, Huntsville, AL 35899 USA; 4 Department of Materials Science and Engineering and Engineering and Inter-University Semiconductor Research Center, Seoul National University, Daehag-dong, Gwanak-gu, Seoul 08826 South Korea

## Abstract

In this paper, we present a unique resistive switching (RS) mechanism study of Pt/TiO_2_/Pt cell, one of the most widely studied RS system, by focusing on the role of interfacial bonding at the active TiO_2_–Pt interface, as opposed to a physico-chemical change within the RS film. This study was enabled by the use of a non-conventional scanning probe-based setup. The nanoscale cell is formed by bringing a Pt/TiO_2_-coated atomic force microscope tip into contact with a flat substrate coated with Pt. The study reveals that electrical resistance and interfacial bonding status are highly coupled together. An oxygen-mediated chemical bonding at the active interface between TiO_2_ and Pt is a necessary condition for a non-polar low-resistance state, and a reset switching process disconnects the chemical bonding. Bipolar switching mode did not involve the chemical bonding. The nature of chemical bonding at the TiO_2_-metal interface is further studied by density functional theory calculations.

## Introduction

As a promising alternative to conventional memories, resistive switching memory has recently attracted significant attention owing to its high speed, low power consumption and excellent scaling potential^1, 2^. Resistive switching (RS) memory cells, usually in a metal-insulator-metal (MIM) structure, exhibit reversible and dramatic changes in their cell resistance between high and low resistance states (HRS and LRS) by electrical biases. Despite numerous studies performed on RS memories and resulting advances in the understanding over the last few decades, there are still many unknowns about the RS mechanism, presenting uncertainties in the eventual scaling limit, read/write speed and device endurance^2, 3^. The difficulties are generally related with the fact that only a small portion of a device is responsible for the overall RS while the portion is usually embedded inside the device structure hindering a direct assessment of the process. *In-situ* transmission electron microscopy (TEM) has significantly extended the understanding of related phenomena at the nano-scale^4–8^, but it actually does not represent a real device condition because of the thin lamellae format of TEM specimen and ultra-high vacuum environment during electrical operation. Therefore, it is an impending task in the field to suggest an additional methodology to further investigate RS mechanism at a nano-scale. This work demonstrates a feasible approach to examine the nano-scale chemico-mechanical change at resistive switching junctions using a rather unusual experimental setup based on a scanning probe technique.

Pt/TiO_2_/Pt memory cell is one of the most widely studied RS systems^9–17^. This system is known to exhibit both thermo-chemical mechanism (TCM) and valence-change mechanism (VCM) depending on the bias application procedure^18^. It is largely accepted that TCM is caused by formation and annihilation of conducting filaments (CFs) within the TiO_2_ layer, and that the CFs are formed in a conical shape with a thinner diameter at the interface where an anodic bias was applied during the electroforming step due to the n-type nature of TiO_2_^19^. The thinner part of the CF near the anodic interface shows repeated rupture and rejuvenation, which is responsible for the TCM-based unipolar resistive switching (URS) while the thicker part of the CF near the other (cathodic) interface remains virtually intact making a (quasi-) ohmic contact with the cathode^1, 3, 8, 9, 20–22^. This makes the anodic interface called the ‘active’ interface in a cell with n-type RS materials. Later, it was found that the chemical identity of CFs in TiO_2_ is Magnéli phase (Ti_n_O_2n−1_ where n is mostly 3 or 4), a highly oxygen-deficient metallic phase, through a rigorous *in-situ* TEM study^8, 23^. On the other hand, VCM relies on electronic barrier modulation at the active interfaces by the migration of oxygen vacancies within the RS film depending on the bias polarity^24^, usually in a localized manner, while the opposite interface maintains (quasi-) ohmic contact. In this case, the electronic switching performance mostly exhibits bipolar resistive switching (BRS) behavior. The operation of BRS in TiO_2_ generally requires asymmetric cell configuration. In the previous studies using a seemingly symmetrical planar Pt/TiO_2_/Pt structure^18^, such asymmetry could be achieved by a URS set (or electroforming) with a higher compliance current and subsequent reset process, which locally rupture the weaker part of CF near the anode. In this configuration, the actual memory cell becomes composed of Pt/retained Magnéli-CF/TiO_2−x_/Pt where the Magnéli-CF/TiO_2−x_ and TiO_2−x_/Pt junctions comprise a (quasi-) ohmic and an active Schottky-like junction, respectively. When the oxygen vacancy concentration within the TiO_2−x_ region increases by applying a negative bias to the nearby Pt electrode with a compliance current small enough not to cause a URS set again, the cell BRS-sets (i.e. becomes a bipolar LRS). An application of the opposite bias would reset the cell. In this case, the remaining Magnéli CF near the opposite side of the cell played the role as the source and reservoir of oxygen vacancies as well as counter-electrode^25^. However, the highly asymmetric physical geometry of the memory cell in the present experimental setup provide the asymmetry needed to induce a BRS as discussed later in this paper.

As described above, RS behavior has been largely attributed to the local change of phase and/or ionic concentrations *within* the RS oxide film. The localized change corresponds to either a formation/annihilation of CFs within the RS film^1–3, 8, 9, 20, 26^ or a localized ionic redistribution (inside the RS film again) causing a modulation of Schottky-like electronic energy barrier at the oxide/electrode interface^1, 3, 21^. In this paper, we present an RS mechanism study of Pt/TiO_2_/M (M: Pt or Au) cells by focusing on the correlation between the adhesive/bonding state at the active interface and the RS behavior, through atomic force microscopy (AFM)-based experimental observations and density functional theory (DFT) calculations. There have been numerous AFM-based studies on RS behavior including CF formation^14, 27–29^, electrochemical reaction-induced RS^21^, coexistence of local and homogeneous RS^30^, and pressure-sensitive RS^31^. However, this experimental setup provided an unprecedented method of explicitly addressing the possible involvement of chemical bonding in RS mechanism. This has not been feasible with large scale memory cells with a standard MIM configuration or even in *in-situ* TEM. This analysis showed that a certain chemical interaction is clearly involved and is actually necessary to maintain the LRS, whereas it is not the case for HRS.

## Results and Discussion

**Figure 1:**
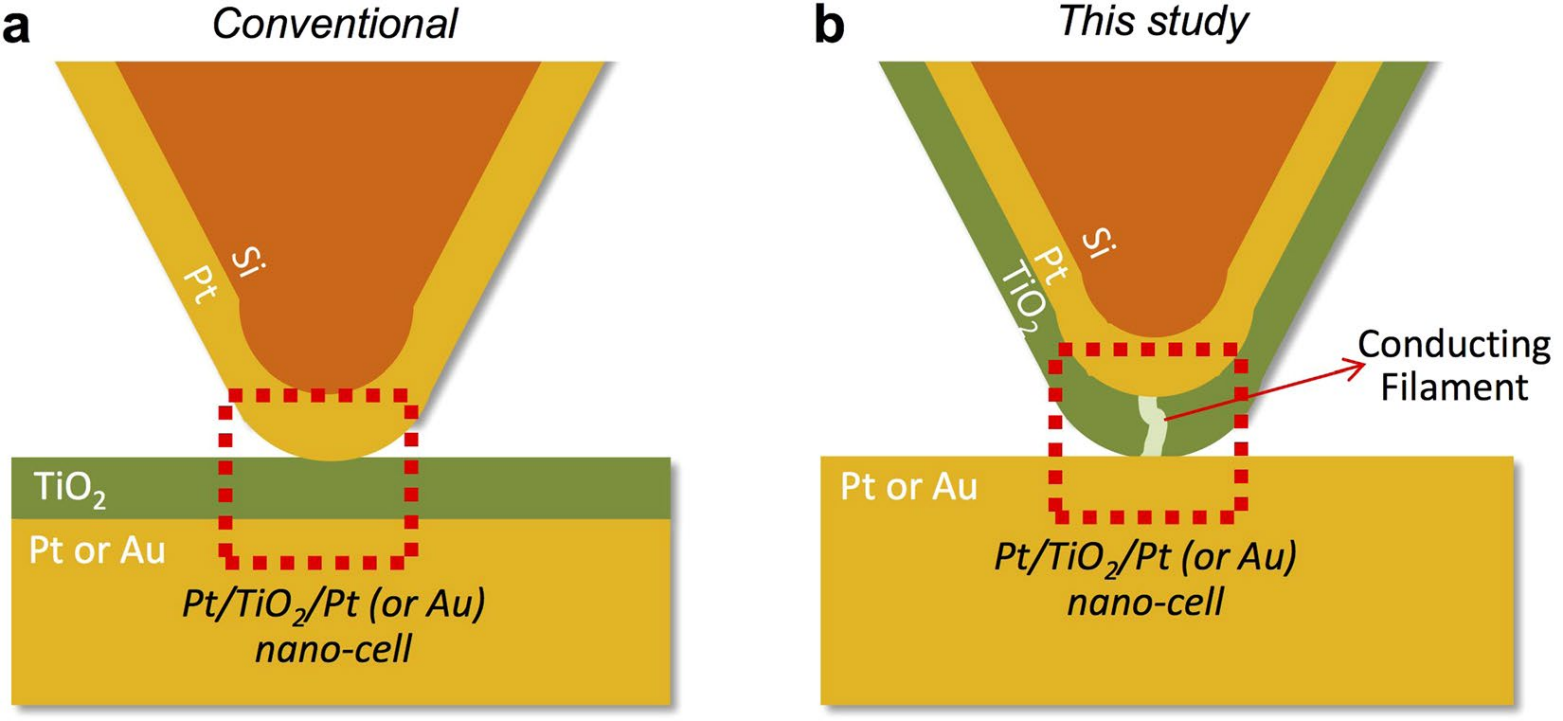
(**a**) The conventional and (**b**) the modified configuration (used for this study) scanning probe-based configuration.

Unlike the conventional AFM configuration shown in Fig. 1a, in our AFM-based setup, an MIM cell is implemented by having a Pt/TiO_2_ coated AFM tip in contact with Pt substrate as shown in Fig. 1b. A similar setup where an oxide is deposited on a metal-coated tip was employed earlier by Cui *et al*. to produce one nanometer resolution electrical path for CAFM^32^. The new configuration is less vulnerable to the uncontrollable drift (1–10 nm/min) in the relative lateral position between the tip and substrate^33^, which is intrinsic to AFM. Even in the new configuration, the location of Pt surface in contact with the Pt/TiO_2_-coated tip changes uncontrollably over time. However, at least the area *within* the TiO_2_ layer affected by any previous switching process will still comprise the nano-cell of measurement wherever the tip is located, which cannot be achieved in a conventional setup. We believe that this is the reason why the critical findings to be presented in this work have not been found in previous studies with conventional setup using Pt-coated AFM tip and TiO_2_ film-coated substrate.

**Figure 2:**
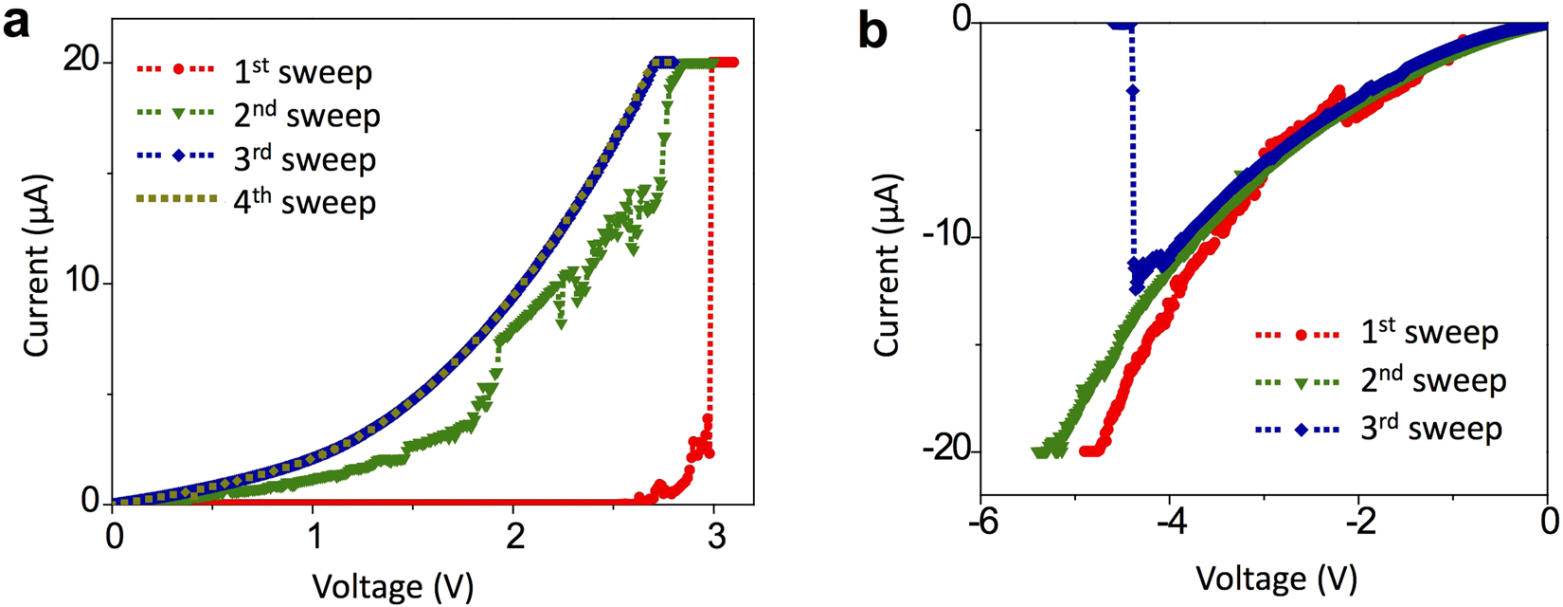
(**a**) *I-V* profiles for an electroforming process of a pristine cell in the new configuration (Pt/TiO_2_ coated tip in contact with a Pt substrate) followed by three potentiodynamic sweeps. Anodic biases were applied to the Pt substrate. (**b**) *I-V* profiles obtained by applying cathodic biases to the Pt substrate after the fourth sweep shown in (**a**). All the sweeps were performed by having the tip on a specific location of Pt substrate without detaching the tip off the substrate.

**Figure 3:**
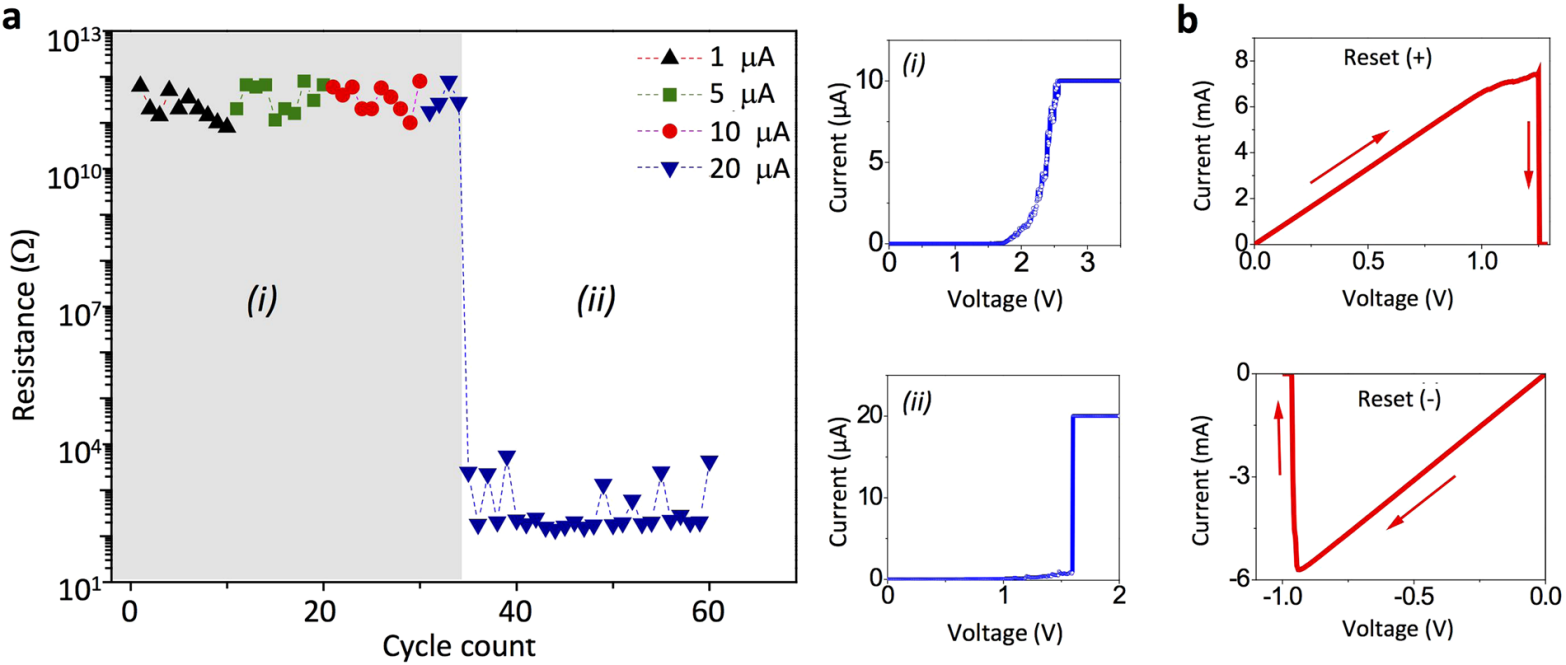
(**a**) Resistances of an as-prepared Pt/TiO_2_/Pt cell measured after a set process with compliance currents of 1, 5, 10 and 20 μA. Each cell resistance measurement was performed after a set process on a new location of the Pt substrate. The upper and lower graphs on the right side show typical *I-V* behaviors before and after the transition into a unipolar switching, respectively. (**b**) A unipolar reset processes with positive (left) and negative (right) bias to the Pt substrate.

Figure 2a shows an initial set of current-voltage (*I-V*) profile obtained in a nanoscale cell formed by having a fresh Pt/TiO_2_-coated tip in contact with a Pt substrate. All the bias polarity is defined positive when the substrate is positively biased with respect to the tip. The electroforming (initial set switching) process is performed with a current compliance of 20 μA. At a voltage of ~3 V, there is an abrupt increase in current. However, the fluctuating behavior in the *I-V* profile obtained immediately after the electroforming process reveals an unstable electrical status of the cell (2^nd^ sweep). However, the overall current level rises with repeated sweeps and stabilized at a non-linear profile (3^rd^ and 4^th^ sweep) characteristic to a Schottky-like contact. During subsequent voltage sweeps with the opposite bias polarity, the current level declined as the sweep proceeds and eventually ended up with an abrupt decrease (Fig. 2b). The behavior (i.e. the gradual increase and decrease of current level under voltage biases with different polarity) was routinely observed in this setup during the initial *I-V* sweeps through a pristine cell. This behavior can be readily understood with mechanistic interpretations of VCM^3, 20^. The experimental configuration itself renders a highly asymmetric memory cell geometry; tiny contact area and high contact resistance between the tip and substrate, and sharp tip shape induce an electric field concentration effect at the contact region, whereas the Pt/TiO_2_ junction within the tip does not feel significant electric field due to its larger area and possibly better interfacial contact. The tip-substrate interface supplies oxygen vacancies to the TiO_2_ film through oxygen evolution reactions (2O^2−^ → O_2_(g) + 4e^−^) when an anodic (positive) bias is applied to the Pt substrate. Since the other interface (i.e. the Pt/TiO_2_ interface within the AFM tip) allows little room for gaseous oxygen in this configuration, oxygen exchange at this interface must be highly limited. Therefore, the Schottky barrier at the TiO_2_-coating/Pt substrate interface becomes lower and the cell resistance decreases. It is noted that oxygen vacancies in transition metal oxides such as TiO_2_ are much more mobile than cations^20^, and it is reasonable to consider the movement of oxygen ions (or oxygen vacancies) as opposed to cations. A bias application in the opposite direction will incorporate oxygen into the lattice through oxygen reduction reactions (O_2_(g) + 4e^−^ → 2O^2−^). For this reaction, abundant gaseous oxygen molecules from the atmosphere or adsorbed oxygen atoms on the Pt surface near the tip can be the source of oxygen ions. Although the presented *I-V* sweeps were not performed in the usual sequence of BRS set and reset switching with alternating bias polarities, it is believed that these sweeps exhibit the nature of BRS because they are well aligned with the VCM governed by ionic migration. The nano-cell enters a URS regime when the *I-V* curve shows an ohmic transport behavior (e.g. a linear *I-V* line) after repeated voltage sweeps with a large compliance current (i.e. 20 µA as will be shown in Fig. 3a). As will be noted later, however, this VCM-type of process does not induce any significant modification of the chemico-mechanical bonding between the TiO_2_-coated tip and Pt substrate.

Figure 3 shows cell resistances, each measured after a set switching process in the Pt/TiO_2_/Pt configuration shown in Fig. 1b by using another as-prepared Pt/TiO_2_-coated tip. The measurements were performed in a dry Ar environment. While the tip was in contact with the Pt substrate, the cell was set-switched by a positive voltage sweep, and the resulting cell resistance was measured by another short voltage sweep. The voltage sweep for the resistance measurement is limited to 10 mV or 500 nA, whichever is reached first, to avoid any appreciable change in the resistive state during the measurement. The resistances were quantified by performing a linear fit to the *I-V* data. After the set switching and subsequent resistance measurement, the tip is pulled away from the substrate, during which a force-distance curve (and thus adhesion force) is obtained. Subsequently, the tip is re-attached to a different location of the substrate before repeating another sequence of set switching, resistance measurement and adhesion force measurement. To investigate the influence of compliance current for set processes on the RS behavior, four different compliance currents (1, 5, 10 and 20 μA) are used (Fig. 3a). For each compliance current of 1, 5, and 10 μA, ten sequences of tip movement, switching, and resistance measurements are performed; for 20 μA, thirty sequences are recorded. By starting with the smallest compliance current (1 μA) among the four, the influence of preceding switching process on the following RS behaviors is expected to be minimized. The transition into a ‘true’ LRS (where an abrupt increase in current occurs during the set switching process and a linear *I-V* relationship is observed in the subsequent voltage sweeps) appeared with the compliance current of 20 μA. Compliance currents less than 20 μA rarely caused the transition to true LRS up to 30 switching cycles. This is consistent with Celano *et al*.’s observation where the compliance current dependency is ascribed to the minimum amount of defects needed for filament formation^34^. Before the transition, the *I-V* profile for set switching typically resembles the graph shown in the upper right side of Fig. 3a. In this case, the current gradually increases with voltage bias without showing a sudden jump in current. This was the case even with a compliance current value of 20 μA up to four cycles (see data with down triangle symbol shown in the shaded area of Fig. 3a), which is consistent with the data shown in Fig. 2. However, from the 5^th^ sweep the cell starts to show a sudden increase in current during a voltage sweep as shown in the lower right graph of Fig. 3a, and the resulting LRS cell showed ohmic conduction behavior with a much higher current level than those in preceding switching cycles. Such cell can be electrically reset regardless of voltage bias polarity as shown in Fig. 3b, exhibiting the URS (or non-polar RS) behavior. Therefore, these observations indicate that the cell now came to have a fully developed Magnéli phase CF penetrating the entire TiO_2_ thickness from the knowledge of TCM mechanism found in TiO_2_-based cells^9^. With the increased number of voltage cycles under high compliance current (20 μA), oxygen evolution occurs around the contact region increasing the concentration of oxygen vacancies in its vicinity. The generated oxygen vacancies are driven toward the opposite Pt/TiO_2_ interface hidden inside the tip and eventually start to nucleate Magnéli-CF(s) at that interface^22^. Therefore, the non-ohmic bipolar LRS and unipolar ohmic LRS are originated from very different physical states in the TiO_2_ layer.

**Figure 4:**
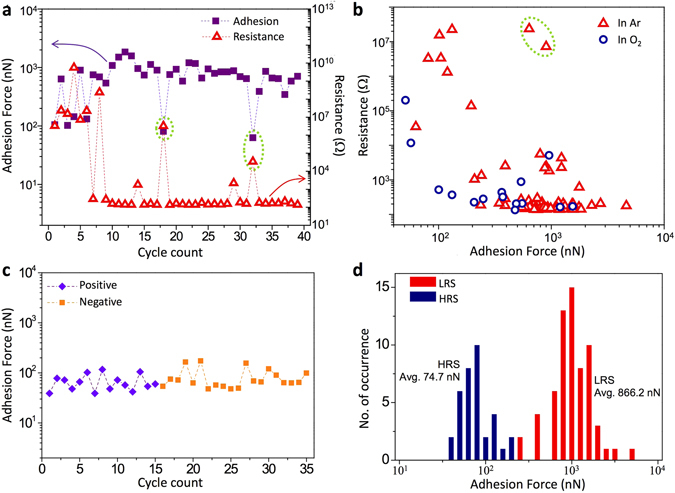
(**a**) The resistances of a Pt/TiO_2_/Pt cell and the corresponding adhesive forces at the active interface (i.e., the interface between the Pt/TiO_2_ coated tip and the Pt substrate). Each set of the resistance and adhesive force is measured sequentially immediately after a set process with a compliance current of 20 μA on a different tip location of Pt substrate. (**b**) Cell resistances and corresponding adhesive forces measured in dry Ar and O_2_ environment. (**c**) Adhesive forces measured right after URS reset processes with positive sweeps (anodic substrate bias; number 1–15) and negative sweep (number 16–35). Numbers do not indicate the measurement sequence. The measured adhesive forces (~100 nN) were significantly lower than those of URS LRS cells (~1 μN). (**d**) Distribution of measured adhesion forces after set and reset processes.

An interesting phenomenon to note is that a set-switched cell loses its unipolar LRS-like state once the tip is detached from the substrate. The cell behaves like a normal HRS cell even when the tip is re-attached onto the same location of the substrate, and is then electrically switchable back to a unipolar LRS by a subsequent set switching. A re-attachment on the exact same location at the atomic scale may not be possible in the setup. However, if metallic CF(s) within the TiO_2_ layer of an LRS cell were simply ‘touching’ the surface of Pt substrate, the cell should maintain its LRS even after a relocation of the tip on the substrate because the surface of Pt substrate should provide a low resistance contact regardless of the location. Since a relocation makes the cell lose its LRS, the contact nature at the tip-substrate interface is presumed to play a crucial role in a URS cell. It is noted that a tip detachment would expose the active interface to a finite amount of oxygen present in the surroundings even under the nominal Ar environment because a continuous flow of Ar may not fully suppress the oxygen activity unlike an ultrahigh vacuum environment. If oxygen in the environment plays a role, the CF(s), especially those in a close vicinity of the exposed surface, may be locally oxidized making a subsequently re-attached cell an HRS regardless of the tip location. As an effort to study the impact of interfacial nature on the RS, interfacial adhesion force was quantified in each RS state. After a newly prepared Pt/TiO_2_ tip is brought in contact with the Pt substrate forming a MIM cell, an anodic bias is applied to the substrate with the compliance current of 20 μA. Then, the resulting resistance and tip-substrate adhesion force are measured. The adhesion force is quantified by obtaining a force-distance curve^35–37^ while the tip is being retracted immediately from the z-directional location where the set switching process and resistance measurement was performed consecutively. After measuring the resistance and adhesion force, the tip is moved to a new location and re-attached to the substrate (see Fig. S1 of on-line Supplementary Information for the sequence). After repeating the sequence described above in a dry Ar environment, the resulting cell resistances and adhesion forces in each cycle are presented in Fig. 4a. It is noted that this pristine cell also changes into a unipolar LRS only after several cycles with a compliance current of 20 μA, as observed in Fig. 3. The adhesion force at the active interface of a cell in unipolar LRS (with the cell resistance <~10^3^ ohms) is mostly higher than 300 nN. However, in some cycles (marked in dotted circles in Fig. 4a and b), the cell resistances are abnormally high, presumably due to a tip-substrate morphological convolution^38^ and/or unexpected contaminants at the contact region. The corresponding adhesive force is found small in this case. Figure 4b reveals the inversely proportional relationship between electrical resistance and corresponding interfacial adhesive force. When there is insufficient adhesive force, the cells are all in HRS even after an intended set process. A strong adhesion formation of > few hundreds of nN is found a necessary condition (as opposed to a sufficient condition) to retain a stable LRS. The adhesion force measurements were performed immediately after a set process with the compliance current of 20 μA in a dry environment, either dry Ar or O_2_, to avoid possible complications by water meniscus (a capillary condensation of water) formed around the tip-substrate contact in atmospheric environment. Figure 4c and d demonstrate another interesting observation that the active interface of a unipolar LRS cell loses its large bonding force after a reset switching. The adhesion force measured after a unipolar reset switching becomes similar to that of a new or a re-attached contact. Furthermore, this is the case regardless of the reset switching polarity. Figure 4c shows adhesion forces, each measured after a unipolar reset process using either a positive (diamond symbol) or negative (square symbol) voltage to the substrate. The tip was first moved to a new location on the Pt substrate, unipolar set switched and then reset switched before each adhesion force measurement. As summarized in Fig. 4d, the measured adhesion forces after a reset process are significantly lower than those of unipolar LRS cells. The average adhesion force is higher in unipolar LRS cells (average: 866.2 nN) than those in HRS (average: 74.7 nN) by more than an order of magnitude. This strongly indicates that bonding status at the active interface is closely coupled with the resistive switching state. In addition, the fact that this behavior occurs regardless of the reset switching bias polarity suggests that the reset switching is less likely an electric field-driven process such as ionic migration and redox reaction. It is more probable to be a pure thermally-activated process such as atomic/ionic diffusion, chemical reaction, phase change and possibly a combination of these. From the fact that the reset current is very large (~7 mA; see Fig. 3b), the CF(s) are likely heated up to a very high temperature^39^, a situation where these process are highly activated. A scanning electron microscopy (SEM) image of a reset tip indeed exhibits a significant change of tip morphology (Fig. S3d). It is also noted that when a tip-surface contact forming a unipolar LRS was relocated by moving the tip to another spot of the Pt surface, the newly formed MIM cell in an HRS showed a low adhesion force (average: 51.3 nN, standard deviation: 7.8 nN from 10 data points obtained in Ar). This is also consistent with the observation of strong correlation between adhesion force and electrical resistance state. The first several cycles of test in Fig. 4a correspond to the intermediate state being transforming to the unipolar LRS, showing a bipolar LRS property. It is interesting to note that this bipolar LRS contact does not bear strong adhesion with the Pt electrode (adhesion force ~100–150 nN). Therefore, the strong adhesion could be achieved only in the case of the unipolar LRS, suggesting its close relationship with the Magnéli-CF formation.

**Figure 5:**
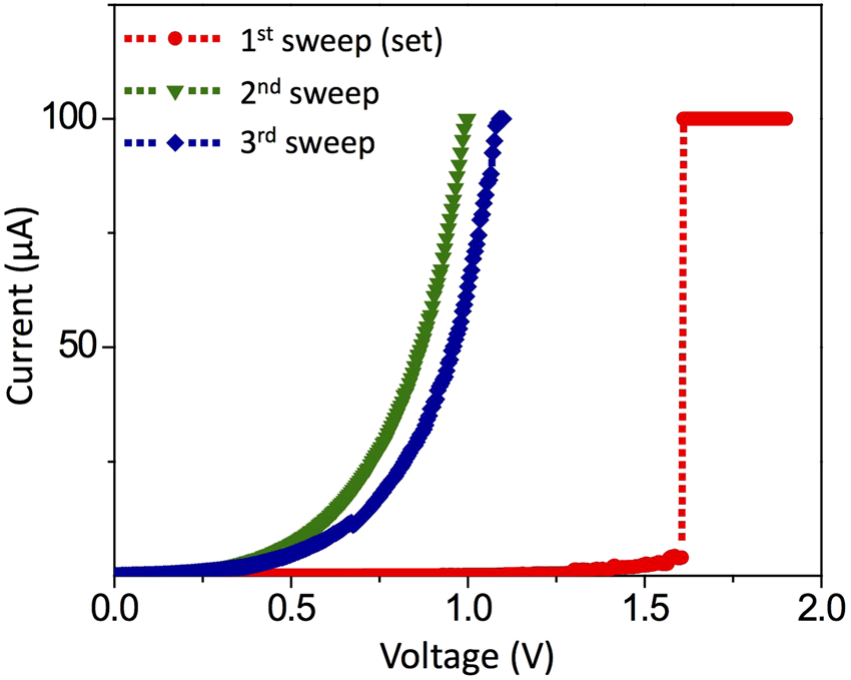
*I-V* graphs for a set process (compliance current = 100 μA) and followed by potentiodynamic sweeps through a scanning probe-based Pt/TiO_2_/Au cell. The Pt/TiO_2_ tip was electroformed on a Pt substrate prior to these sweeps.

As a comparison study, RS processes are also performed on an Au substrate. In this additional test, the Pt/TiO_2_ tip was first unipolar set-switched on a Pt substrate and moved to an Au substrate. Under this circumstance, it was expected that the tip has a well-established CF(s) within the TiO_2_ layer. Figure 5 shows a set process achieved by a few time repeated *I-V* sweeps. It is first noted that the Pt/TiO_2_/Au cell shows low current suggesting that the cell is in HRS despite the TiO_2_ layer was previously unipolar set-switched. Therefore, this is also consistent with the aforementioned strong positive correlation between adhesion force and LRS. The cell set-switched again at ~1.6 V. Although an abrupt surge in current is observed during a set switching with a very high compliance current of 100 μA, the subsequent sweep did not show an ohmic current flow suggesting that the unipolar LRS is not recovered. (Note that the compliance current for a set process used on Pt substrates was 20 μA). In this case, the tip-substrate adhesion forces were <100 nN after set processes, which are significantly smaller than those of unipolar LRS cells formed on a Pt substrate and similar to that of a fresh contact. This indicates that the bonding needed to render a unipolar LRS on Pt does not readily form at the interface with Au. This observation is also in agreement with previous reports on Au/TiO_2_-based RS^40–42^, in which only bipolar switching behavior was observed. This may be due to a higher free energy of oxide formation on Au^13^. Alternatively, a stable nanoscale Magnéli phase (the chemical identity of CFs in TiO_2_)^8^ may not be formed on an Au surface unlike Pt.

Attractive interfacial interactions can be categorized into chemical force, electrostatic force and van der Waals interaction^43^. The mechanism of interfacial bonding formation during a set process is currently unknown, but the electrostatic and van der Waals interactions can be readily excluded from the bonding mechanism candidates in our RS system. The usual adhesion force in a LRS cell spans from 0.2–5 μN as shown in Fig. 4d, mostly around 1 μN. However, the typical bonding energy of van der Waals interaction is ~0.01–0.1 eV^44^, which is equivalent to a value at least 2–3 orders of magnitude smaller than the measured quantity given the cell geometry (assuming a 100 nm^2^ of contact area at the tip-substrate interface). The electrostatic interaction is also simply ruled out because there would be little electrostatic force formed between the tip and substrate when appreciable amount of current can flow between the two. In our experiments, however, bonding forces are measured to be even higher when the contact forms an LRS (that is, when little electrostatic force can be induced). For these reasons, the high attractive force at the TiO_2_/Pt interface of an LRS cell is attributed to a chemical bonding.

Now, we check if CFs can form a chemical bonding with Pt substrate by density functional theory (DFT) calculations. A single crystal Magnéli phase Ti_4_O_7_ was used to model CFs and examined if it could form a stable interface structure with either Pt or PtO_2_. The single crystal model should be reasonable as the Magnéli phase observed in experiment has several nm in size^8^ where polycrystalline structure is less dominant. The surface formation energy was calculated for low-index facets of *Fm* $\bar{3}$ *m*-Pt and *Pmnn*-PtO_2_ to identify the most stable surfaces of the Pt and PtO_2_ substrates. For PtO_2_, only Pt/O-termination was examined excluding Pt-termination to emphasize the distinct surface characteristic of PtO_2_, distinguished from Pt. The total electronic energy of slab structures of Pt or PtO_2_, with 15–20 Å of thickness and 20 Å of empty space between slabs, were obtained from DFT calculations. Then, the surface formation energy of facet *i* was defined as ${\gamma }_{i}=({E}_{{\rm{slab}}}^{i}-{E}_{{\rm{bulk}}})/2{S}_{i}$, where ${E}_{slab}^{i}\,{\rm{and}}\,{E}_{bulk}$ are the energy of slab with surface facet *i* and the bulk structure with the same volume of the slab, respective, and *S*_*i*_ is the corresponding surface area. The results are given in Table 1. The result indicates that the (111) facet of Pt and the (011) facet of PtO_2_ are the most stable, which are compatible with previous studies^45–48^.

**Table 1:** The calculated surface formation energy or interface formation energy.

Material	Facet/Interface	*γ*(J/m^2^)
Pt	(100)	1.90
	(110)	1.91
	(111)	1.47
PtO_2_	(100)	2.00
	(010)	1.39
	(001)	4.49
	(110)	1.54
	(101)	1.47
	(011)	1.07
	(111)	2.69
Ti_4_O_7_	(001)	1.26
Ti_4_O_7_-Pt	Special	3.49
Ti_4_O_7_-PtO_2_	Special	0.29

**Figure 6:**
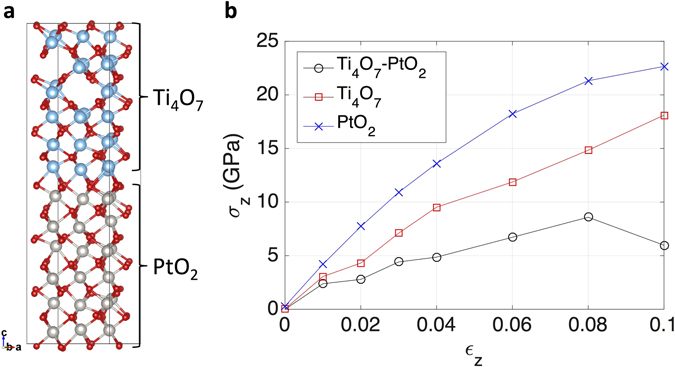
(**a**) Illustration of an interface between Ti_4_O_7_ and PtO_2_ (interface area: 52.5 Å). The blue, grey, and red spheres indicate Ti, Pt, and O, respectively. (**b**) The stress-strain relationship during elongation in z-axis for three different materials.

‘Special’ indicate the facets to have the Ti sub-lattice aligned with the Pt sub-lattice as illustrated in Fig. 6.

The interfaces between Magnéli phase Ti_4_O_7_ and (111) Pt and (011) PtO_2_ were modeled and the interface formation energy was obtained accordingly. We choose the (001) facet of Ti_4_O_7_ and aligned its Ti sub-lattice with Pt sub-lattice of Pt or PtO_2_ across the interface as shown in Fig. 6a, which comprises a coherent interface with a maximized stability. DFT calculations were conducted for the constructed interfaces and their bulk components. The corresponding interface formation energy was defined as ${\gamma }_{{\rm{int}}}=(E-{E}_{{\rm{bulk}}1}-{E}_{{\rm{bulk}}2})/2{S}_{{\rm{int}}}$, where *E* is the energy of a constructed interface, *S*_int_ is the interface area, and *E*_bulk1_ and *E*_bulk2_ are the energies of the two bulk components. The results are also given in Table 1. The positive interface formation energy indicates that the constructed interface is unstable compared to its separated state. While the interface formation energy of Ti_4_O_7_–PtO_2_ is positive, it is as low as 0.29 J m^−2^ (or 0.018 eV Å^−2^), which is accessible by thermodynamic fluctuation at room temperature, and hence implies that the Ti_4_O_7_–PtO_2_ interface is meta-stable. However, a comparison between the interface formation energy and the summation of the surface formation energy of each component has higher significance than the calculated interface energy itself. The comparison indicates that the Ti_4_O_7_–PtO_2_ interface is more stable than the system where Ti_4_O_7_ and PtO_2_ surfaces are separated, whereas the Ti_4_O_7_–Pt interface is less stable than their separated surfaces. These calculation results are well aligned with the experimental findings; when the Ti_4_O_7_ CF-containing TiO_2_ film of the tip is in a mere physical contact with the Pt (or Au) surface, the high interface energy will not allow them to form intimate contact. Therefore, the cell remained in HRS. However, when the anodic bias is applied to the Pt substrate, which drags additional oxygen atoms from nearby TiO_2_ (or even Ti_4_O_7_ CF), the contact region of the Pt electrode will be oxidized to PtO_2_. Electrical current flowing through the contact region would induce Joule heating, providing the energy needed to overcome the activation energy for the formation of a stable chemical bonding between the Ti_4_O_7_ CF-containing TiO_2_ film and Pt (PtO_2_) substrate. Although the interface energy between the (anatase) TiO_2−x_ and Pt has not been calculated, it can be reasonably expected that it may be comparable to the Ti_4_O_7_-Pt case. Therefore, the non-formation of strong adhesion in the case of bipolar LRS mentioned above can also be understood from the same line of consideration.

We also obtained a theoretical stress-strain relationship in the meta-stable Ti_4_O_7_–PtO_2_ interface (Fig. 6b) to assure the formation of a chemical bonding. The supercell was elongated in the direction perpendicular to the interface, and the ionic relaxation was conducted while retaining the elongated supercell shape and volume. This tensile test was also performed for bulk state Ti_4_O_7_ and PtO_2_ for a comparison. The stress was then obtained as a byproduct of DFT calculations during the ionic relaxation. The resulting stress-strain relationships are displayed in Fig. 6b. It is clearly shown that the stress-strain relationship of the Ti_4_O_7_–PtO_2_ interface is different from those of bulk Ti_4_O_7_ and PtO_2_; the stress for the Ti_4_O_7_–PtO_2_ interface deflects off the increasing trend at the strain of ~10% while those for Ti_4_O_7_ and PtO_2_ continue to increase. This result also indicates that the bonding at the interface is chemical, not just a superposition of the chemical bonds of Ti_4_O_7_ and PtO_2_.

From the aforementioned study, we can conjecture the following scenario of RS process. First, a sufficient electroforming process (and some additional set processes) will form well-defined Magnéli phase CF within TiO_2_ RS film with a (quasi-) ohmic contact with the cathodic interface. The major portion of well-defined CF well inside the tip region does not change its phase or shape significantly during subsequent switching processes. A unipolar RS starts only after the active interface (anodic interface) forms the aforementioned chemical bonding with the Pt anode. (Before the chemical bonding formation, it may still switch in BRS mode based upon VCM). During a unipolar set switching, the electric field will be concentrated at (or in a very close vicinity of) the anodic interface, and the interface will be heated up to a very high temperature for the interface to quickly relax into a stable (or a meta-stable) configuration, aligning their atoms/ions as predicted by thermodynamics. As soon as a stable interface enabling a high flux of electronic transport is knitted, a unipolar LRS is achieved, which would instantaneously spread the intense electric field (highly concentrated in the very close vicinity of anodic interface) throughout the whole CF length. This would quench the highly heated interface and thus forms a solid chemical bonding. While the importance of a chemical bonding formation at the active interface for resistive switching is well justified by the aforementioned study, there are still many unknowns about the nature of bonding. Further study on the nature of active interface and its impact on RS are intended for subsequent reports.

## Conclusions

A series of observations described in this report suggests that the bonding nature at the ‘active interface’ largely determines the RS state once a URS is activated. The following lists a brief summary of the observations made in this study: 1) When an AFM-based Pt/TiO_2_/Pt cell (formed by a contact between a Pt/TiO_2_ coated tip and a Pt substrate) is in a unipolar LRS, the adhesive force at its active interface is higher than those found in an HRS cell and bipolar LRS by more than an order of magnitude on average; 2) once a tip-substrate interface comprising an LRS cell is mechanically separated, the cell becomes an HRS even after the tip is promptly re-attached to the same location of the substrate. The cell then behaves like a normal HRS cell and can be electrically set to an LRS again; 3) when an LRS cell is reset-switched to an HRS, the active interface returns to the initial low adhesive state. This is the case regardless of the reset switching polarity; 4) Furthermore, the nature of the adhesion at the active interface of an LRS cell is from a chemical bonding (not a van der Waals interaction or electrostatic force) and the adhesion is also found strongly dependent on the oxygen activity and back electrode material. These observations collectively indicate that the URS behavior is closely coupled with the interfacial/adhesive nature at the active interface. These also suggest that the URS mechanism of metal oxide-based RS cells can be more completely understood through research focused on the chemical reactions involving oxygen activity at the active interface.

## Methods

### Tip and Substrate Fabrication

An MIM configuration was implemented by having a Pt/TiO_2_ coated AFM tip in contact with a Pt or Au-coated substrate in a commercial AFM system (Model 5500, Agilent Technology). Pt-coated substrate was fabricated by sputter-depositing ~100 nm-thick Pt film on a SiO_2_/Si wafer at an Ar pressure of 20 mTorr. A commercial Au-coated SiO_2_/Si wafer (Sigma-Aldrich) was used as the Au substrate. A 20 nm TiO_2_ thin film was deposited on a commercial Pt-coated probe (ElectriMulti75-G, Ted Pella, Inc.) by atomic layer deposition (ALD; Savannah 100, Cambridge NanoTech) at 250 °C using Tetrakis (dimethylamido) titanium (IV) and de-ionized water as the Ti precursor and the oxygen source, respectively. X-ray diffraction (XRD; PANalytical X’Pert PRO, Co Kα radiation) analysis of TiO_2_ deposited on a Pt substrate showed that the TiO_2_ film was a polycrystalline anatase structure (Fig. S2, on-line Supplementary Information).

### AFM Measurement

All electrical characterization was performed by a semiconductor parameter analyzer (HP 4145B) in a two electrode setup in which voltage bias was applied to the substrate while the tip was grounded. Both the tip and substrate were enclosed in a glass chamber with a volume of ~2,600 cm^3^ to control the gas environment. Dry environment was obtained by feeding in dry oxygen (99.99%) or Ar gas (99.99%) into the chamber at 1 liter per min (lpm) for >30 min before the start of each measurement. Gas flow of 1 lpm was maintained throughout the entire measurement for ensuring the dry gas environment. To study the adhesive force between the Pt/TiO_2_ tip and the metal-coated substrate, force-distance curve^49^ was performed before and after switching processes. To eliminate possible contaminants, tips were plasma-cleaned (Harrick Plasma) in air for 1 min and etched by ethanol for 10 min, and metal-coated substrates were cleaned by ethanol before being introduced into the dry gas-enclosed AFM setup. The nominal tip radius of commercial Pt-coated tip is ~25 nm and the force constant of the cantilevers is ~3 N/m, but there is a wide variance in the force constant values (reportedly 1–7 N/m) leading to a corresponding variance expected in pressing force. After a TiO_2_ deposition by ALD, the usual tip radius spanned 35–40 nm.

### Computational Method

The electronic energy calculation was conducted based on the DFT as implemented in Vienna Ab-initio Software Package (VASP)^50–53^. Spin-polarized generalized gradient approximation with Perdew–Burke–Ernzerhof parameterization for the exchange-correlation functional was used^54, 55^. The volume and shape of supercell were allowed to change during the ionic relaxation, and a high cutoff energy of 520 eV was used to exclude the effect of Pulay stress.

## Electronic supplementary material

Supplementary information
