# Supplementary material for: Interfacial chemical bonding-mediated ionic resistive switching
Source: Sci Rep. 2017 Apr 28;7:1264. doi: 10.1038/s41598-017-01493-x (PMC5430901; doi:10.1038/s41598-017-01493-x)
Supplement: Supplementary file 1 — Supplementary information [file 41598_2017_1493_MOESM1_ESM.pdf]

Supplementary Information

Interfacial chemical bonding-mediated ionic resistive switching

Hyeongjoo Moon[1], Vishal Zade[1], Hung-Sen Kang[1], Jin-Woo Han[2], Eunseok Lee[3*], Cheol Seong Hwang[4], and Min Hwan Lee[1*]

[1]School of Engineering, University of California, Merced, CA 95343, USA
[2]Center for Nanotechnology, NASA Ames Research Center, Moffett Field, CA 94035, USA
[3]Department of Mechanical and Aerospace Engineering, University of Alabama, Huntsville, AL 35899, USA
[4]Department of Materials Science and Engineering and Engineering and Inter-University Semiconductor Research Center, Seoul National University, Daehag-dong, Gwanak-gu, Seoul, 08826, South Korea
[*]mlee49@ucmerced.edu, eunseok.lee@uah.edu

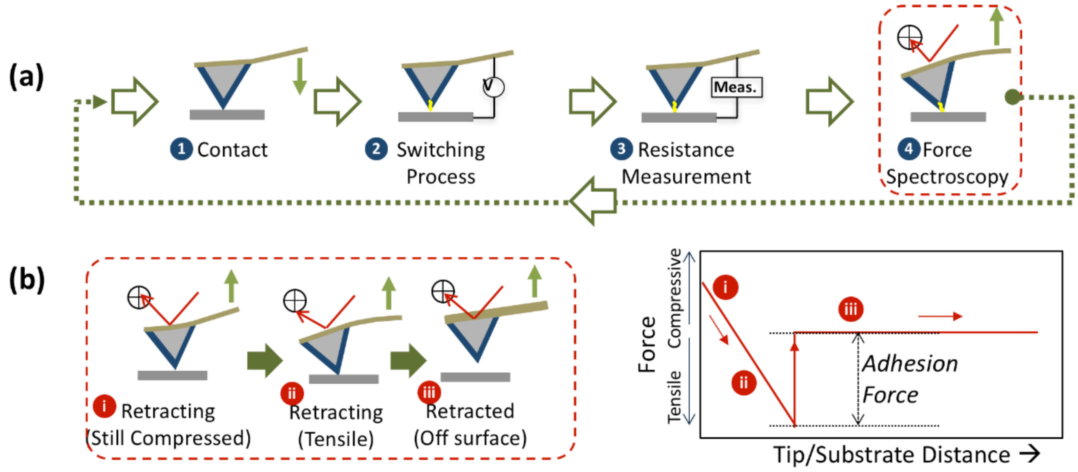

Figure S1. (a) A schematic diagram delineating the electrical resistance and adhesion force measurement processes. Immediately after a set-switching measurement and a resistance measurement, the tip is pulled off the surface during which a force-distance curve is acquired to quantify the adhesion force. (b) The sequence of AFM force spectroscopy to quantify adhesive forces between the tip and substrate (left) and an illustrative force-distance graph (right).

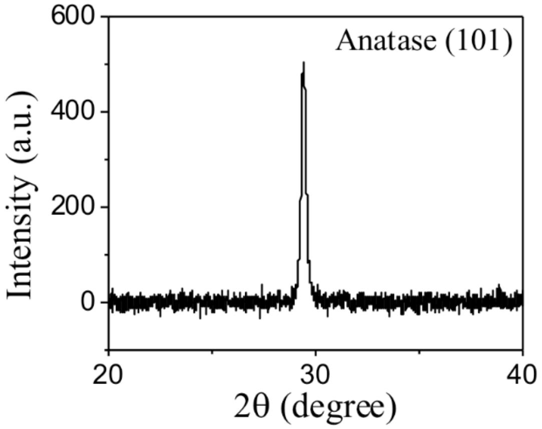

Figure S2. XRD pattern indicating the anatase phase of TiO_2. Sample was prepared by depositing TiO_2 by ALD on a sputtered Pt.

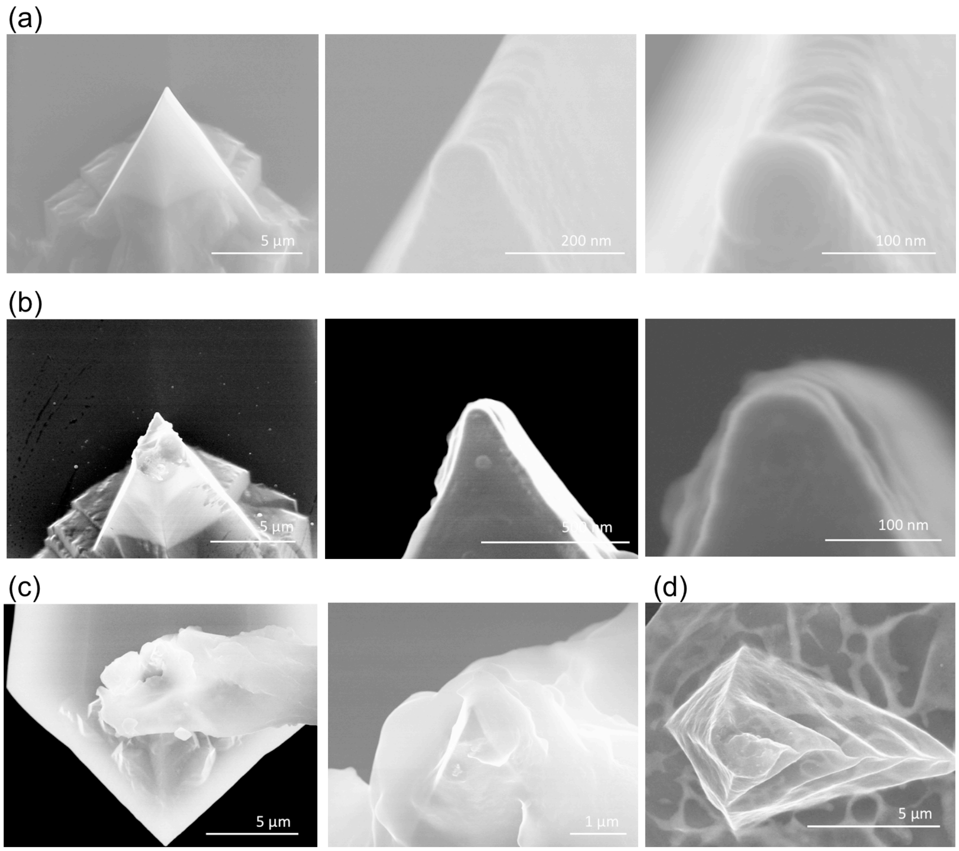

Figure S3. (a-b) SEM micrographs around AFM tip apex with different magnifications before (a) and after an URS set-switching with a compliance current of 20 μA (b). (c) shows another set of images with two different magnifications around a set-switched tip (with a 20 μA compliance current). Here, the Pt back-electrode is peeled off its underlying Si substrate and adhered to the tip probably due to a strong tip-

substrate adhesion. (d) A top-view image of a tip after a reset switching (with a reset current of ~4 mA) showing a significant change in tip morphology and surface texture. The tip is directed toward the viewing angle.
